# Transcranial direct current stimulation for treatment-resistant obsessive-compulsive disorder: report on two cases and proposal for a randomized, sham-controlled trial: Estimulação transcraniana por corrente contínua para o transtorno obsessivo compulsivo resistente ao tratamento: relato de dois casos e proposta de um ensaio clínico randomizado e controlado por *sham*

**DOI:** 10.1590/1516-3180.2016.0155010716

**Published:** 2016-09-26

**Authors:** Renata de Melo Felipe da Silva, André Russowsky Brunoni, Eurípedes Constantino Miguel, Roseli Gedanke Shavitt

**Affiliations:** I MD. Doctoral Student, Obsessive-Compulsive Spectrum Disorders Program, Department and Institute of Psychiatry, Hospital das Clínicas (HC), Faculdade de Medicina da Universidade de São Paulo (USP), São Paulo, SP, Brazil.; II MD, PhD. Director, Service of Interdisciplinary Neuromodulation (SIN), Department and Institute of Psychiatry, Hospital das Clínicas (HC), Faculdade de Medicina da Universidade de São Paulo (USP), São Paulo, SP, Brazil.; III MD, PhD. Full Professor of Psychiatry, Obsessive-Compulsive Spectrum Disorders Program, Department and Institute of Psychiatry, Hospital das Clínicas (HC), Faculdade de Medicina da Universidade de São Paulo (USP), São Paulo, SP, Brazil.; IV MD, PhD. Director, Obsessive-Compulsive Spectrum Disorders Program, Department and Institute of Psychiatry, Hospital das Clínicas (HC), Faculdade de Medicina da Universidade de São Paulo (USP), São Paulo, SP, Brazil.

**Keywords:** Obsessive-compulsive disorder, Electric stimulation therapy, Electrode, Electric stimulation, Transcranial direct current stimulation., Transtorno obsessivo-compulsivo, Terapia por estimulação elétrica, Eletrodos, Estimulação elétrica, Estimulação transcraniana por corrente contínua.

## Abstract

**CONTEXT AND OBJECTIVE:**

Neuromodulation techniques for treating obsessive-compulsive disorder (OCD) have expanded through greater understanding of the brain circuits involved in this disorder. Transcranial direct current stimulation (tDCS), a non-invasive technique, has been studied as an alternative for treatment-resistant OCD. We describe the design of a clinical trial using tDCS for OCD and report on the outcomes from two patients with primary OCD who were resistant to cognitive-behavioral therapy and to selective serotonin reuptake inhibitors, and who received tDCS in an open manner during the training phase for the study procedures.

**DESIGN AND SETTING:**

Methodological description of a clinical trial using tDCS for treatment-resistant OCD at a university hospital; and a report on two cases.

**METHODS:**

The proposed study is randomized, sham-controlled and double-blind. Forty-four patients will be randomized to either active or sham intervention. The active intervention consists of applying an electric current of 2 mA, with the cathode positioned in the region corresponding to the supplementary motor cortex (bilaterally) and the anode positioned in the deltoid. The primary outcome will be the reduction in baseline YBOCS (Yale-Brown Obsessive Compulsive Scale) score at the end of week 4. The secondary outcomes will be depression and anxiety symptoms. Genetic markers, cortical excitability and neurocognitive performance will be investigated.

**RESULTS:**

The first patient showed significant improvement, whereas the second remained symptomatic after four weeks and after six months. tDCS was well tolerated.

**CONCLUSION:**

tDCS for treatment-resistant OCD merits randomized controlled trials that test its effectiveness.

## RESUMO

**CONTEXTO E OBJETIVO:**

Técnicas de neuromodulação para o tratamento do transtorno obsessivo compulsivo (TOC) vêm se expandindo com o melhor entendimento dos circuitos cerebrais envolvidos no transtorno. A estimulação transcraniana por corrente contínua (ETCC), uma técnica não invasiva, vem sendo estudada como alternativa para o TOC resistente aos tratamentos convencionais. Descrevemos o desenho de um ensaio clínico utilizando a ETCC para o TOC e relatamos os desfechos de dois pacientes com TOC primário resistentes à terapia cognitivo-comportamental e aos inibidores seletivos de recaptura da serotonina, que receberam ETCC de modo aberto durante a fase de treino dos procedimentos do estudo.

**TIPO DE ESTUDO E LOCAL:**

Descrição metodológica de um ensaio clínico utilizando ETCC para o TOC resistente num hospital universitário e relato de dois casos.

**MÉTODOS:**

O estudo proposto é randomizado, controlado por *sham* e duplo-cego. 44 pacientes serão randomizados para intervenção ativa ou simulada (*sham* ). A intervenção ativa consiste em aplicar uma corrente elétrica de 2 mA, com cátodo posicionado na região correspondente à área cortical motora suplementar (bilateralmente) e ânodo posicionado no deltoide. Desfecho primário: redução do escore inicial da escala YBOCS (Yale-Brown Obsessive Compulsive Scale) ao final da quarta semana. Desfechos secundários: sintomas depressivos e ansiosos. Marcadores genéticos, excitabilidade cortical e performance em testes neurocognitivos serão investigados.

**RESULTADOS:**

O primeiro paciente apresentou melhora significativa, enquanto o segundo permaneceu sintomático ao término das quatro semanas e após seis meses. A ETCC foi bem tolerada.

**CONCLUSÃO:**

A ETCC para o tratamento do TOC resistente merece ensaios clínicos randomizados que testem sua efetividade.

## INTRODUCTION

Obsessive-compulsive disorder (OCD) is an often-chronic and potentially disabling condition with a prevalence of 2-3% in the general population,^1^^,^^2^^,^^3^ characterized by obsessions (unwanted repetitive thoughts and images) and compulsions (repetitive behavior usually adopted to alleviate the discomfort elicited by the obsessions). OCD is frequently associated with other psychiatric conditions such as mood and anxiety disorders.^2^ The impairment relating to OCD affects personal, social and occupational functioning, and it thus leads patients and their families to poorer quality of life, to an extent that is similar to what is seen in relation to schizophrenia and mood disorders.^4^^,^^5^^,^^6^^,^^7^

The first-line treatments for OCD include use of the serotonin reuptake inhibitor (SRI) clomipramine, selective serotonin reuptake inhibitors (SSRIs) and cognitive-behavioral therapy (CBT). However, about one third of patients fail to experience significant improvement after these first-choice treatments.^8^ Augmentation of SRI use with CBT or antipsychotics has been reported to increase the response rates, but a considerable number of patients remain significantly ill.^9^

Therapeutic alternatives involving neuromodulation have been considered for cases of treatment-resistant OCD. There is evidence that OCD is associated with dysfunction in the frontal-striatum-pallidum-thalamic circuitry, including the dorsolateral prefrontal cortex (DLPFC), orbitofrontal cortex (OFC), medial prefrontal cortices, supplementary motor area (SMA), anterior cingulate gyrus and basal ganglia.^10^^,^^11^ Neuromodulation techniques targeting these routes may be invasive, such as deep brain stimulation; or noninvasive, e.g. repetitive transcranial magnetic stimulation^12^ and transcranial direct current stimulation (tDCS).^13^

tDCS consists of applying a direct electric current across two relatively large electrodes: the cathode and the anode. This current is able to penetrate the skull and reach the cerebral cortex, thereby modifying the neuronal membrane resting potential^14^^,^^15^ and modulating the neuronal firing rates. In fact, tDCS increases cortical excitability without inducing an action potential.^16^ It has shown promising results for depression and schizophrenia, with a favorable safety and tolerability profile.^17^^,^^18^

With regard to OCD, Narayanaswamy et al. reported two cases of SSRI-refractory patients who received 20 sessions of tDCS at 2 mA, with 40% improvement.^19^ In another open-label study, eight treatment-resistant OCD patients received 10 sessions of tDCS at 2 mA. Five of them showed at least 25% improvement in baseline Y-BOCS (Yale-Brown Obsessive Compulsive Scale) scores and three of them showed at least 35% improvement.^20^^,^^21^ These small heterogeneous open studies suggest that tDCS may be effective for OCD.

## OBJECTIVE

Thus, the aim of the present paper was to report on the outcomes of two patients whose primary disorder was OCD. They had been unresponsive to cognitive-behavioral therapy and/or SSRIs or clomipramine, and they received 20 sessions of tDCS during the pilot phase of a randomized clinical trial (RCT). First, we describe the methodology and then we assess the efficacy and safety of tDCS for treating OCD. The intervention delivered to the two patients reported on was identical to that described below.

## METHODS

### Design

This is a randomized, double-blind, sham-controlled trial. After randomization, all subjects will receive 20 daily sessions of 30 minutes duration of either tDCS or a sham intervention for four weeks (Mondays through Fridays). Follow-up assessments will take place at weeks 4, 6, 9 and 12. All subjects and staff members will be blind to the treatment condition, except for the registered nurse and an attending physician at the Neuromodulation Unit who will conduct the sessions and are not members of the research team. The project has been approved by our institution's Research Ethics Committee (1.015.347) and is registered at ClinicalTrials.gov (NCT02743715).

### Sample and eligibility

There are no controlled clinical trials that assess tDCS for treating OCD. Therefore, the sample size calculation will be based on the repetitive transcranial magnetic stimulation (rTMS) for OCD studies available in the current literature, due to the proximity of the intervention model, sample characteristics and outcome measurements. Thus, based on one meta-analysis,^22^ which found that active rTMS was more favorable than sham intervention, with Hedges g of 0.59 (z = 2.73; P = 0.006) for a two-tailed P of 0.05 and a power of 95%, the total sample size is 33 subjects. Taking an attrition rate of approximately 30%, we estimated that the final sample size should be 44 patients. The patients will be recruited from the OCD Spectrum Disorders Program at the Institute of Psychiatry, University of São Paulo Medical School.

The inclusion criteria are: age 14-65 years; a primary DSM-5^23^ diagnosis of OCD and a baseline Yale-Brown Obsessive-Compulsive Scale (Y-BOCS) score greater than or equal to 16.^21^ The presence of psychiatric comorbidities will be allowed, provided that OCD is the primary disorder. Up to two failed previous pharmacological treatment methods (SSRIs and SSRIs combined with anti-psychotics) and CBT will be allowed. Subjects who have failed these three treatment methods will not be admitted into the study. Current use of medication will be permitted, as long as the doses have been stable for at least six weeks.

The exclusion criteria comprise presence of severe suicidal ideation (structured plan for suicide or attempted suicide within the past four weeks); bipolar disorder; substance abuse/dependence; schizophrenia or psychotic disorders; dementia; pregnancy; or specific contraindications for tDCS (metal plates on the head or anatomical abnormalities).

### Staff

A registered nurse trained in the procedures of tDCS sessions will be the person to take care of the patient from his/her arrival at the facility until discharge. She will accompany the patient through the tDCS sessions and will be allowed to operate the tDCS device under medical supervision. These two members of the team will be the only non-blind staff in the study. The other members of the team will be two clinical psychiatrists, who will be responsible for recruitment, computerized randomization and the medical appointments over the course of the study; and a research assistant with a degree in psychology who will be responsible for the baseline and 12^th^ week assessments.

### Interviews

The following instruments will be used: demographic and socioeconomic (ABIPEME)^24^ forms, personal and family medical histories, and the following questionnaires: Dimensional Yale-Brown Obsessive Compulsive Scale (DY-BOCS),^25^ Y-BOCS, Sensory Phenomena Scale (USP-SPS),^26^ Beck Depression Inventory (BDI),^27^ Beck Anxiety Inventory (BAI),^28^ Brown Assessment of Beliefs Scale,^29^ Clinical Global Impression-Improvement subscale (CGI),^30^ World Health Organization Quality of Life assessment (WHOQOL-100),^31^ and Structured Clinical Interview for DSM-4 (SCID-I).^32^ It should be noted that the SCID for DSM-5 is not currently available or validated in the Portuguese language. Therefore, our strategy will be to have the subjects interviewed by trained clinicians using the SCID-IV and then to make diagnoses by means of the DSM-5 criteria, using clinical experience to move between the two instruments.

### Intervention

The intervention will consist of stimulation by means of a direct electrical current to the cathode, which is positioned in the area corresponding to the bilateral supplementary motor cortex. The anode is positioned in the left deltoid (neutral region). Our choice for the location of the electrodes was based on the findings of Senço et al.^33^ In their systematic review, it was found that placing the cathode on the pre-SMA and the anode in the extra-cephalic area seemed to activate most of the areas relating to OCD, based on computerized head modeling analysis on electrode positioning in transcranial magnetic stimulation and deep brain stimulation trials on OCD.

The current strength will be 2 mA on a surface of 25 cm^2^, applied in 30-minute daily sessions for 20 consecutive weekdays. In the sham group, the device will be turned off after 30 seconds of active stimulation. This is a blinding method that has been used previously to induce the same sensations as in active stimulation, like mild tingling on the skin.^34^

### Outcomes

The primary outcome will be the reduction in baseline YBOCS scores at the end of the fourth week. The secondary outcomes will include measurements of depression and anxiety symptoms. To evaluate patients treated in an open manner, a follow-up will be conducted six months afterwards in order to assess the long-term effect of the intervention.

### Tolerability

Any adverse effects from tDCS will be measured by means of the Systematic Assessment for Treatment Emergent Effects (SAFTEE) and the tDCS adverse events questionnaire.^35^^,^^36^

### Case reports

A preliminary, open phase of the proposed trial was conducted to enable implementation of the study routines, and to test the tolerability of the procedures and train the staff in the study procedures. Two patients with severe treatment-resistant OCD received the tDCS intervention as described above in the intervention section. It should be noted that, differing from the inclusion criteria of the proposed study, the two patients described below had histories of failure in more than two SSRI trials. Both of them tolerated the study procedures well. The first patient maintained his severe symptoms, whereas the second showed significant improvement, which was maintained over time.

- Case 1: This patient was a 37-year-old married businessman with incomplete higher education. He had been diagnosed with OCD, comorbid social phobia and generalized anxiety disorder at the age of 16 years. His illness was characterized by a need for symmetry, ordering, counting and arranging. The symptoms had worsened over time and, despite his own efforts, he was unable to control them, which had led to impairment of his relationships and social life. A lot of effort was required from him to meet his objectives at work. Therapeutic failures with SSRIs and side effects from drugs added to his suffering. He had never required hospitalization and had no family history of OCD. At the time of admission, he was taking fluoxetine (100 mg/day). His YBOCS scores were 38 (0% improvement) at baseline, 38 (0%) at four weeks, 38 (0%) at twelve weeks and 31 (18% improvement), six months after completing the intervention, with no significant changes in depression or anxiety symptoms.
- Case 2: This patient was a 31-year-old unemployed man with incomplete higher education who was living with his parents. He had been diagnosed with OCD at the age of 19 years, with comorbid depressive symptoms and a lifetime history of psychotic (paranoid) symptoms, for which he had required hospitalization. He had received electroconvulsive therapy for depressive symptoms and had used marijuana and cocaine. His OCD was characterized by contamination/cleaning and aggression symptoms. As a result of the symptoms, the patient could not keep a job or complete university, and he depended on his parents. Previous treatments with SSRIs and combinations of SSRIs and neuroleptics were unsuccessful. He had completed 20 sessions of CBT, with partial improvement that was not maintained over time. At the time of recruitment he was taking escitalopram (30 mg/day), risperidone (2 mg/day), clozapine (400 mg/day) and valproic acid (500 mg/day). His YBOCS scores were 40 at baseline, 33 (17% improvement) at 12 weeks and 18 (55% improvement), 6 months after completion of tDCS. There were 50% reductions in both the BDI and the BAI scores.

## DISCUSSION

The aims of this study were to describe the methods of a controlled trial of tDCS for treatment-resistant OCD and to report on the outcomes from two patients with treatment-resistant OCD who were treated in an open manner with tDCS. One patient showed minimal improvement over time, with a 19% reduction in baseline YBOCS score, six months after completion of tDCS, whereas the other patient showed a significant improvement at completion of the treatment, which was maintained six months later (45% reduction in baseline YBOCS score).

In line with the results from our second patient, three previous open studies presented promising results for tDCS. Bation et al. reported improvements of at least 25% in five out of their eight patients and 35% in three of them.^20^ Narayanaswamy et al. reported a 40% improvement in two cases and D'Urso et al. reported a 30% improvement in one subject.^19^^,^^37^ With regard to improvement of depression and anxiety symptoms, which were prominent in our second patient, the same observation was reported by Narayanaswamy et al.^19^ Unlike in our study, Bation et al. excluded patients with additional psychiatric diagnoses, whereas D'Urso et al. did not assess the outcomes of mood or anxiety.^20^^,^^37^

It should be noted that the extant studies differ in the positioning of the electrodes. Bation et al. located the cathode over the left OFC and the anode over the right cerebellum; Narayanaswamy et al. placed the anode on the left pre-SMA/SMA and the cathode over the right supraorbital area; and D'Urso et al. placed the active electrode on the pre-SMA and the neutral electrode on the right deltoid.^19^^,^^20^^,^^37^ Therefore, the ideal positioning of the electrodes is still a matter of debate.

## CONCLUSIONS

In conclusion, our observation on the two cases reported here was that a trial of 20 consecutive, 30-minute sessions of tDCS at 2 mA was well tolerated. By running a randomized, double-blind, sham-controlled trial, we intend to further clarify several questions regarding the effectiveness of tDCS for treatment-resistant OCD patients, including factors associated with the short and long-term response and the role of psychiatric comorbidities in the outcomes.
